# Role and exploitation of underground chemical signaling in plants

**DOI:** 10.1002/ps.5507

**Published:** 2019-07-08

**Authors:** Alessandra Guerrieri, Lemeng Dong, Harro J Bouwmeester

**Affiliations:** ^1^ Plant Hormone Biology Group Swammerdam Institute for Life Sciences (SILS), University of Amsterdam Amsterdam the Netherlands

**Keywords:** rhizosphere, root exudates, signaling molecules, agriculture, pest management

## Abstract

The soil ecosystem is composed of a mixture of living organisms and non‐living matter as well as the complex interactions between them. In the past 100 years or so, agricultural soil ecosystems have been strongly affected by agricultural practices such as tillage and the use of pesticides and fertilizers, which strongly affect soil nutrient composition, pH and biodiversity. In modern pest management, however, the focus is gradually shifting from crop production through agricultural practices to soil ecosystem protection. In this review we discuss how the underground chemical signals secreted by plant roots play a role in keeping the soil ecosystem in balance and how they affect plant fitness by shaping the root biome, increasing nutrient availability, promoting symbiosis, and attracting beneficial organisms and repelling harmful ones, including other plants. We review a number of fascinating cases, such as signaling molecules with dual, positive and negative, functions and bacterial quorum sensing mimicking molecules. Finally, examples of how these compounds can be exploited in modern pest management are reviewed, and the prospects for future developments discussed. © 2019 The Authors. Pest Management Science published by John Wiley & Sons Ltd on behalf of Society of Chemical Industry.

## 1 INTRODUCTION

Through the domestication of plants and animals, humans laid the foundation for our modern‐day society.^1^ The concomitant development of agricultural practices, intended to increase field productivity, brought about profound alterations in soil structure and soil biodiversity, with detrimental effects.^2^ For example, agricultural practices have been shown to negatively affect biodiversity in the soil food web.^3^, ^4^ To bring this development to a halt, in 2015 the United Nations formulated a Sustainable Development Goal to achieve improved food security with better product quality, but with less influence on the soil ecosystem.^5^, ^6^, ^7^ In order to achieve this goal, a better understanding of the soil ecosystem is needed. Soil is a highly complex entity in which a multitude of interactions between organisms and the soil matrix take place. All these factors and interactions together constitute the soil ecosystem, the functioning of which determines the availability to plants of mineral nutrients and other abiotic resources, as well as the presence of biotic agents, all of which potentially influence plant fitness.

Plants are not just passengers in all these processes, but actively shape their environment using chemical communication. In recent years, more and more attention has been paid to the interaction of plants with their belowground environment, mainly focusing on the narrow zone of soil that surrounds the plant root and is called the rhizosphere.^8^ In this review, we emphasize the functional role of the chemical compounds that are secreted by plants into this rhizosphere and that affect the physiochemical properties of this root zone or act as chemical signals for other organisms. We discuss the importance of these chemical signals for shaping the soil ecosystem. Finally, the possibilities of using these chemicals as leads for the development of new agrochemicals and/or to develop integrated pest management (IPM) strategies, both of which can be used to achieve a more sustainable agriculture, are discussed.

## 2 THE SOIL ECOSYSTEM AND ITS IMPORTANCE FOR AGRICULTURE

Soils are complex entities resulting from the interaction of many factors: climate, organisms in the soil, soil matrix and topography.^9^ Soils provide the substrate for nature as well as agriculture and in both cases the soil represents a soil ecosystem. This soil ecosystem is not just a mixture of living and non‐living matter, but also encompasses the complex interactions between these components. A better understanding of the relationships between this living and non‐living matter is key in grasping the consequences of changes in the sometimes delicate balance that often occurs in agriculture.^2^

The soil ecosystem is particularly important for agriculture since it contributes to the decomposition of organic matter and litter, which plays a major role in resource recycling,^10^ nutrient retention and uptake by the plant, water regulation and biogeochemical cycling.^11^, ^12^ All these processes together potentially enrich the soil with mineral nutrients and redistribute the organic matter that comes from plant residues,^13^ increasing soil health and fertility and thus improving crop yield. In addition, soil organisms influence many aspects of the plant, from belowground to aboveground. For example, root microbiota can help plants with the uptake of the micro‐ and macronutrients necessary for their growth, such as nitrogen^14^, ^15^ and phosphorus,^16^ and thus prevent their loss through greenhouse gas emission and leaching or immobilization, respectively.^17^ Root‐associated microorganisms can also impart resistance to pathogens^18^ or act as rhizoremediators, phytostimulators and stress controllers, as will be discussed below.^19^, ^20^

Although these soil organisms potentially represent a powerful resource to improve the agricultural soil ecosystem and crop yield, agricultural practices such as tillage, crop rotation, fertilizer and pesticide application and monoculture profoundly affect the soil fauna and microbial community composition, which usually results in a loss of biodiversity and/or decrease in biomass.^4^, ^21^ The latter is mainly caused by tillage as it alters soil microhabitats and interrupts the life cycle of organisms with a long life span and larger body size such as earthworms, mites and enchytraeids.^4^ Le Guillou *et al*.^21^ confirm that tillage also has a large effect on bacterial and fungal diversity and evenness, and destroys mycorrhizal hyphal networks, which results in decreased phosphorous uptake by and availability to the plant.^22^

The impact of the application of mineral fertilizers is complex. Geisseler and Scow^23^ showed that as a result of the higher plant productivity there is more organic material secreted into the soil in the form of root exudates and residues, which stimulates growth of the microbial community that uses this pool of carbon as the main resource. On the other hand, fertilizers, in particular urea and ammonium, decrease the soil pH, which negatively affects microorganisms and reduces the solubility of other nutrients. As far as pesticides are concerned, Asad *et al*.^24^ suggest that in most cases herbicides increase organic acid exudation, which results in the attraction of acidophilic microorganisms and stimulates denitrification. According to Srinivasulu and Ortiz,^25^ pesticides, in low concentrations, stimulate bacterial populations, but are detrimental in combination with fungicides at higher doses. As underlined by Satapute *et al*.,^26^ the major concern about pesticides is their accumulation in the field, which influences not only soil organisms, but directly also soil properties such as pH and nutrient content.

Some of the agricultural practices mentioned here are fundamental farming principles that are applied to improve crop productivity and yield. On the other hand, they are among the factors that seem to profoundly affect organisms and processes that are critical for the long‐term stability of the soil ecosystem. It will be of fundamental importance to better understand these complex relationships such that the soil ecosystem can be more optimally geared for a sustainable productive agriculture. Critical here is also to understand the interaction between plants and the soil and the organisms living in it through root‐exuded chemical compounds and how agriculture can benefit from this.

## 3 HOW DO PLANTS INTERACT WITH THE SOIL ECOSYSTEM?

Lyon and Wilson^27^ in 1921 were the first to show that crops such as maize, oats, peas and broad beans grown in sterile nutrient solution release organic nitrogen into the medium. These authors focused only on nitrogen‐containing molecules, but more recent studies have revealed that root exudates contain also carbon‐based and organic as well as inorganic compounds.^28^ The latter include ions, CO_2_, protons, H_2_, free O_2_ and water,^29^ while organic compounds represent the majority of the molecules produced and secreted by roots. They are released into the soil in a process called rhizodeposition and collectively called rhizodeposits, and include enzymes, amino acids, organic acids, sugars, proteins, mucilage and secondary metabolites such as phenolics (mainly benzenoids, flavonols, lignins and anthocyanins), isoprenoids (sterols and terpenoids), alkaloids and sulfur‐containing compounds like glucosinolates.^30^

Lyon and Wilson^27^ linked the presence of organic nitrogen in the growing medium to the sloughing off of root cap cells since there was no direct evidence that these compounds were released in any other way. However, rhizodeposits are not only derived from the release of dead root cells, but are also actively secreted by the plant itself. Annual crops translocate about 21% of the total fixed carbon to the roots, and grasses about 33%.^31^ From the carbon transported belowground in annual crops and grasses, 3% and 5%, respectively, are released into the soil through rhizodeposition, while 8% and 12% of assimilated C is lost as root‐derived CO_2_ and the remaining percentage is allocated to the root system itself.^31^ Since plants are investing a substantial amount of carbon into the production and secretion of these metabolites, an important question is what the fitness benefit is of this process.

**Figure 1:**
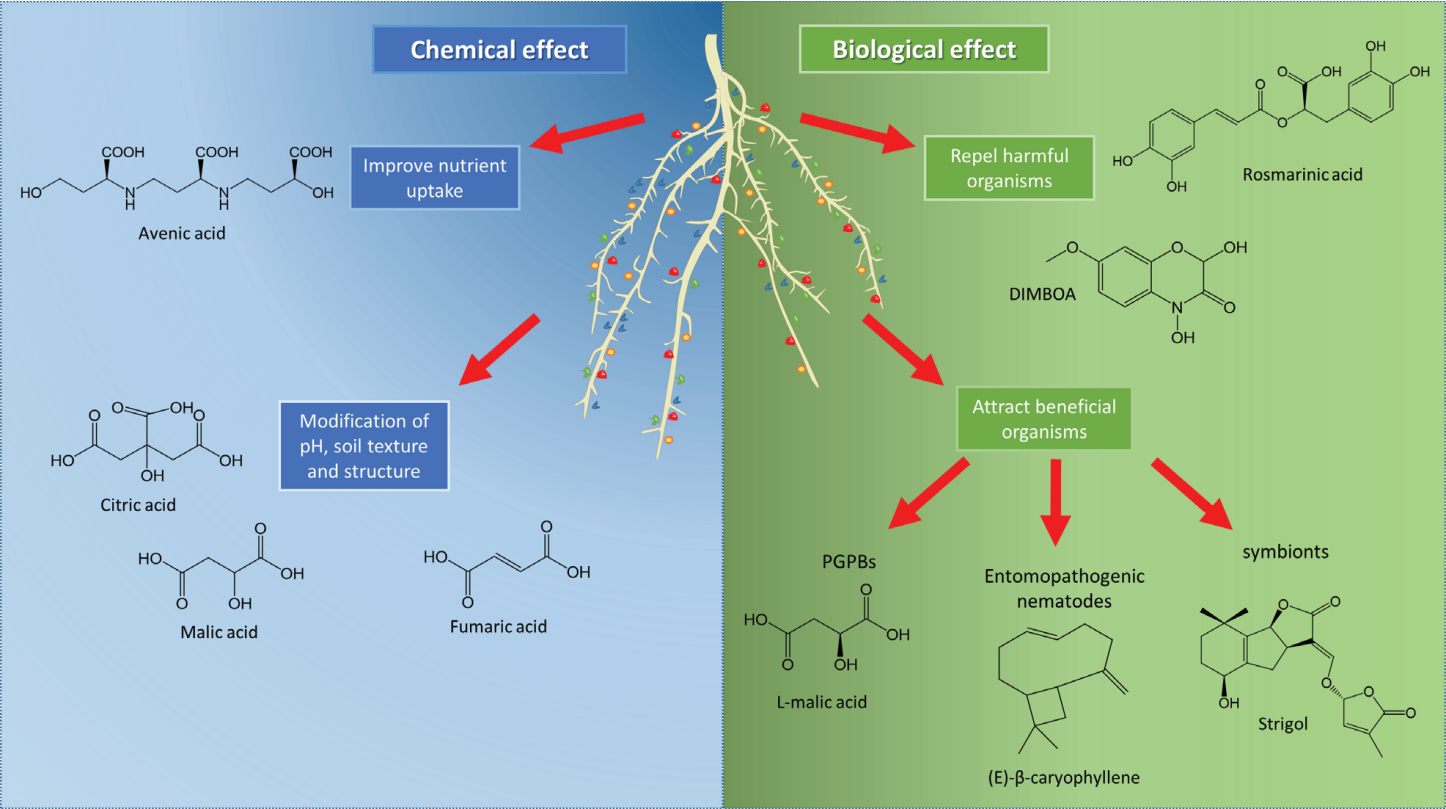
Schematic representation of the different roles of root exudate compounds (represented by differently colored shapes around the roots). Root exudate compounds are divided into two categories: on the left molecules that exert chemical effects by improving nutrient uptake or modifying soil properties, on the right molecules that exert a biological effect, repelling harmful organisms or attracting beneficial ones.

Root exudates have both a chemical and a biological effect on the surrounding environment, with roles in nutrient acquisition^32^ and the interaction with soil organisms (Fig. 1). The biological effect of the root exudate is the chemical signaling between plants and the other organisms living in the soil. Plants can alter their rhizosphere biome, recruiting protective organisms upon pathogen or insect attack^33^ or attracting useful microbes and fungi to improve nutrient uptake, as will be discussed in more detail below. The chemical effect of root exudates is linked to the complex physico‐chemical characteristics and nutrient availability of soils that affect plant physiology.^34^ Plants can alter the rhizosphere environment by modifying soil properties such as pH, texture and soil structure in order to improve the physical conditions for root penetration, and nutrient and water uptake.^35^, ^36^ For instance, as shown by Read *et al*.,^35^ plants release phospholipids as surfactants that reduce the root tip surface tension and facilitate root growth through the soil. Organic acids and sugars that are present in root exudates influence soil texture by increasing soil dispersion and aggregation, respectively.^36^ Soil dispersion might increase nutrient release by soil particles, while aggregation might result in a more stable structure around the roots.

## 4 ROLES PLAYED BY PLANT SIGNALING MOLECULES IN THE SOIL ECOSYSTEM

### 4.1 Nutrient acquisition (pH and phytosiderophores)

Phosphorus is an essential element for plant growth. It is part of membrane lipids, phosphate‐containing molecules such as ATP and NADPH, and nucleic acid building blocks.^37^ Phosphorus availability is mainly influenced by soil pH: in acidic soils phosphorus reacts with iron and aluminum, while in alkaline or calcareous soil it reacts with calcium, making this element inaccessible for plant uptake.^38^ Agriculture relys on a non‐renewable source of this nutrient that will soon be depleted: rock phosphate.^39^ Another important element for plant growth is iron, which is a co‐factor of many enzymes and is involved in chlorophyll biosynthesis. Despite the abundance of iron in the soil, it is not readily bioavailable due to its low solubility, especially in calcareous soil.^40^ Some secondary metabolites and organic acids released in the root exudates by plants are able to solubilize these two nutrients or to modify the soil pH in order to increase their solubility and mobility.^41^ For example, organic acid anions such as citrate, malate and fumarate are released, especially by dicots and non‐graminaceous monocots, in order to acidify the rhizosphere, making Fe, together with P and other micronutrients, more available.^41^, ^42^ Plants can also chelate and solubilize these nutrients with other strategies, for example by releasing phenolic compounds or using phytosiderophores.^32^, ^41^ This last strategy is mostly used by graminaceous plants for Fe uptake, but also by many bacteria that are able to solubilize and chelate Fe, therefore competing with plants.^32^ On the other hand, plants assimilate iron also from bacterial siderophores,^43^ and according to Jin *et al*.,^44^ under iron‐deficiency, red clover alters its root microbial community by exuding phenolic compounds in order to promote colonization by siderophore‐secreting bacteria.

### 4.2 Symbiosis

For the uptake of the macronutrients phosphorous and nitrogen, plants also cooperate – and communicate – with microorganisms, such as arbuscular mycorrhizal (AM) fungi and Rhizobia. An interesting example of the communication between plants and these microorganisms is presented by the strigolactones (SLs). SLs are powerful inducers of germination of parasitic plants of the Orobanchaceae, which then infect their host to obtain assimilates and nutrients.^45^, ^46^ SLs were later also shown to be plant hormones regulating processes such as shoot branching and root architecture.^46^ In 2005, Akiyama *et al*.^47^ applied root exudate fractions of *Lotus japonicus* to the AM fungus *G. margarita*, and showed that the most active fraction inducing hyphal branching contained the SL 5‐deoxystrigol. Intriguingly, under phosphate deficiency, plant species such as tomato, maize and sorghum produce more SLs. SLs induce hyphal branching in germinating spores of AM fungi,^47^ which facilitates the initiation of a symbiosis – with over 80% of all land plants – in which fixed carbon from the plant is exchanged for minerals absorbed from the soil by the fungus.^46^ Despite the essential role of SLs in the initiation of the symbiosis, the mechanisms underlying the perception by the fungus has not been elucidated yet and the fungal receptor(s) are still unknown.

The second well‐studied case is that of the symbiosis between legumes and the Gram‐negative soil bacteria defined as ‘Rhizobia’^48^ that can colonise the roots of legumes and induce the formation of specific structures, called root nodules.^49^ Within the nodules, bacteria differentiate into bacteriods and through nitrogen fixation convert atmospheric nitrogen (N_2_) into a reduced form and make it available to the host.^50^ Flavonoids that are secreted by legumes have been shown to act as chemo‐attractants for Rhizobia and induce the first step in the process required to establish the symbiosis: the induction of the secretion of the bacterial nodulation (nod) factors.^51^ For example, Peters *et al*. found that 3′,4′,5,7‐tetrahydroxyflavone (luteolin) secreted by *Medicago sativa* induces *nodABC* expression in *Rhizobium meliloti* and that this is required for the induction of the early host responses, cortical cell division and root hair curling.^52^ This symbiosis occurs under low nitrogen conditions and is stimulated under those conditions by the (enhanced) production and secretion of specific flavonoids by the host root.^53^ Just as for the strigolactones, the flavonoids are not only perceived by the beneficial Rhizobia but are also used as cues by pathogens. For example, in 1992 Morris and Ward^54^ discovered that the isoflavones daidzeina and genistein, exuded by soybean roots, are a chemo‐attractant to the zoospores of *Phythophthora sojae*, a fungal pathogen.

### 4.3 Molecules attracting beneficial organisms

In addition to beneficial symbiotic organisms such as AM fungi and Rhizobia, a growing body of literature shows that other microorganisms, collectively called plant growth promoting bacteria (PGPBs), can also play important roles in the growth, development and survival of plants.^55^ These PGPBs (and also certain non‐AM fungi) help plants to overcome abiotic stresses such as salinity^56^, ^57^ and drought,^58^ and increase growth and plant fitness in soils contaminated with heavy metals.^59^, ^60^ Despite the fact that many genera of bacteria have been identified as PGPBs, and their roles for the whole plant well studied, for most of these plant‐microbe relationships it is still unclear if and how plants recruit them and, if so, if and which compounds in the root exudates are responsible for this.

Already in 1984, Harwood *et al*.^61^ showed that aromatic acids are chemo‐attractants for *Pseudomonas putida*, one of the most important and versatile PGPBs. Some strains of this PGPB produce the plant hormone indoleacetic acid (IAA), which enhances the development of the root system in, for example, canola seedlings,^62^ helping the plant to get better access to soil nutrients.^63^ Maize is another crop that attracts this PGPB. Neal *et al*.^64^ demonstrated that the benzoxazinoid 2,4‐dihydroxy‐7‐methoxy‐2*H*‐1,4‐benzoxazin‐3(4*H*)‐one (DIMBOA) producing wild‐type maize attracted significantly higher numbers of *P. putida* than the DIMBOA‐deficient *bx1* mutant.^64^ Sometimes bacteria are also attracted by a blend of chemo‐attractants secreted by plants, for example the PGPBs *Bacillus amyloliquefaciens* and *Pseudomonas fluorescens* are attracted by amino acids and organic acids produced by cucumber and tomato roots.^65^, ^66^

These examples suggest rather low specificity of the attraction but it has been demonstrated that the root exudate composition of plants changes upon pathogen attack and that this results in the attraction of beneficial bacteria. For example, an infection with the foliar pathogen *Pseudomonas syringae* pv *tomato* resulted in the higher production and secretion of l‐malic acid in *Arabidopsis* root exudates, which resulted in turn in the recruitment of the PGPB *Bacillus subtilis*.^67^ The interaction with this beneficial bacterium triggers induced systemic resistance (IRS) as well as the promotion of plant growth, thus giving protection against infection by *P. syringae*.^67^

PGPBs are not the only beneficial microorganisms present in the soil that can be attracted by root exudates and can establish a successful relationship with plants. Biocontrol fungi (BCF) such as *Trichoderma* spp. are also an important biological factor in the control of plant diseases. *Trichoderma* spp. interact directly with soil pathogens using efficient mechanisms such as mycoparasitism, antibiotic production and competition for nutrients.^20^ A recent study from Lombardi *et al*.^68^ showed that tomato plants exposed to abiotic and biotic stress attracted the germ tubes of *Trichoderma* spp. Although the attractants were not identified, the authors suggested that peroxidases and oxylipins may be involved.

### 4.4 Molecules repelling harmful organisms

Through their root system plants interact not only with beneficial organisms, but also with pests and pathogens. For a soil‐borne pathogen, roots are the first entry point into the plant and for soil dwelling insects and arthropods roots represent an important food source. Through root exudates plants can release defensive compounds, either upon attack (induced defense; phytoalexins) or constitutively (phytoanticipins).^69^ These molecules act as a first line of defense against pathogen infection.^70^ Some of these molecules change their activity in the rhizosphere when they are modified by other organisms. Benzoxazinoids, for example, attract *Pseudomonas putida* to maize, and are biodegraded by soil microorganisms to phenoxazinones, which have antifungal and antibacterial properties.^64^

Phenolics and terpenoids often have strong antimicrobial and antiherbivory properties.^70^ Rosmarinic acid, for example, which is secreted by the roots of sweet basil (*Ocimum basilicum*), challenged by the pathogenic fungus *Pythium ultimum* showed antimicrobial activity against rhizosphere microorganisms.^71^ Lanoue *et al*.^72^ showed that barley, when attacked by *Fusarium*, produces antifungal phenolic compounds such as *t*‐cinnamic acid, which is biosynthesized *de novo* and released by the plant root.^72^ *Arabidopsis*, however, constitutively produces the diterpenoid rhizathalene A, a semi‐volatile phytoanticipin that is involved in defense against soil herbivores.^73^ The mechanisms underlying the antifungal and antimicrobial activity of such compounds are largely unknown, with some exceptions. Rosmarinic acid, for example, disrupts fungal cell wall integrity and thus prevents fungal growth. In bacteria such as *Pseudomonas aeruginosa*, the same compound leads to the proliferation of cell division, causing DNA condensation and altered morphology.^71^

Another strategy used by plants to control the rhizosphere microbiome, in particular the bacteria growing around their root system, is producing molecules that can mimic bacterial communication molecules called quorum sensing (QS) signals. QS is based on the synthesis, detection of and response to bacterial QS signals such as the homoserine lactones (HSLs) that accumulate in the environment as the bacterial population increases.^74^, ^75^ The QS response triggers the expression of genes, amongst others involved in biofilm formation and virulence. In *Agrobacterium tumefaciens*, for example, the transfer of the tumor‐inducing plasmid into a plant cell is activated by HSLs.^76^ Sensing of and interfering with the QS signaling molecules of pathogenic bacteria potentially provide a fitness benefit to plants. So far, few QS mimicking compounds have been discovered and characterized. Corral‐Lugo *et al*.^74^ discovered that rosmarinic acid is not only produced after microbial infection as an antimicrobial, as already discussed above,^71^ but also interferes with the QS‐induced activation of virulence factors in *P. aeruginosa*. Binding studies showed that rosmarinic acid binds with high affinity to the *P. aeruginosa* RhlR regulator activating the signaling cascade that normally is activated by the bacterial *N‐*butanoyl‐homoserine lactone (C4‐HLS), stimulating biofilm formation and virulence factors.^74^ This activation of the bacterial QS mechanism when the population density is still low has been proposed as a defense strategy, but this hypothesis is still under debate. This principle could in theory also be used in agriculture to interfere with pathogen QS signaling in the soil and hence prevent plant diseases. Indeed, Pérez‐Montaño *et al*.^77^ suggest that rice and bean produce HSL‐mimicking signals that enhance or interfere with the biofilm formation of two plant‐associated bacteria. They hypothesize that beneficial microorganisms are recognized by the plant and are then stimulated by the plant by the secretion of HSL‐mimicking signals, while pathogens would be controlled also through QS‐mimicking molecules. In both cases, plant QS‐mimicking compounds showed a higher affinity for the bacterial QS receptors than the bacterial QS molecules.^74^ Therefore, a low concentration of the QS‐mimicking molecules can already outcompete the bacterial signal.

### 4.5 Tritrophic interactions

In the case of biotic stresses caused by insects and soil‐borne pathogens, plants can produce and exude defensive phytoalexins, as discussed above, or produce signaling compounds to attract protective microbes^78^ or (other) natural enemies of the pest that is attacking the plant. These so‐called tritrophic interactions are particularly important and useful in agriculture since the organisms involved can be applied in IPM as biological control agents.^79^ The central mechanism in tritrophic interaction is that – upon attack – plants produce infochemicals that diffuse through the soil matrix and are perceived by enemies of the attacking pest.^80^ Maize plants under attack from maize corn rootworm (*Diabrotica virgifera virgifera*), one of the most important maize pests that is invading Europe, release the sesquiterpene (*E*)‐β‐caryophyllene from their roots.^81^ (*E*)‐β‐caryophyllene attracts the entomopathogenic nematode *Heterorhabditis megidis*, which can efficiently parasitise corn rootworm. Ali *et al*.^82^, ^83^ showed that the hybrid citrus Swingle (*Citrus paradisi* Macf. x *Poncirus trifoliate* L. Raf.) attracts the entomopathogenic nematode *Steinernema diaprepesi* when attacked by the root‐feeding weevil *Diaprepes abbreviates*, through production of a C12 terpene cue, pregeijerene. Interestingly, pregeijerene also attracted a phytopathogenic nematode: *Tylenchulus semipenetrans*. Just as the strigolactones and flavonoids described above, this represents another example of a pathogenic species that has hijacked a compound with a positive function for the plant (a synomone or allomone) and thus converted it into a pest attractant (kairomone).

A special case of attracting enemies of your enemy, as recently suggested by Eppinga *et al*.,^84^ is the recruitment by plants of soil organisms that damage other plants competing for the same resources. The authors hypothesized that the exotic invasive plant species *Ammophila arenaria* accumulates local pathogens that are not adapted to it in order to damage neighboring local plant species. This hypothesis is supported by a study of Mangla *et al*.,^85^ who showed that root exudates from the invasive species *Chromolaena odorata* increased the presence of the pathogenic fungus *Fusarium semitectum* at the expense of native plants.

### 4.6 Allelopathy

Plants are not just competing with other organisms, but also with other plants, either conspecific or with different species. Allelopathy is a long‐known mechanism by which the fitness of plants is increased through the release of allelochemicals, compounds that can interfere with growth or other vital processes, such as germination, in competing plant(s) (species) either directly or upon degradation or transformation in the soil. Well‐studied examples of this are phenolics, alkaloids and terpenoids.^86^ Although allelochemicals can be produced in different parts of the plant, here we focus on the ones secreted by roots.

For example, *Ligularia cymbulifera*, a native Chinese herb that is expanding into grasslands and causes a decrease in forage grass yield in the Hengduan Mountains in China, secretes phytotoxic sesquiterpenes to outcompete other plant species, causing cell death in the root tips and consequently inhibiting root elongation.^87^ Phenolic compounds have several beneficial roles in the soil, as discussed above, but can also cause autotoxicity in perennial species such as alfalfa and clover that are mainly used as feed for livestock. In general, phenolic compounds interfere with hormone activity, membrane permeability, photosynthesis and synthesis of organic compounds,^86^ and are mostly produced under nitrogen shortage. Another well‐studied allelochemical is sorgoleone, present in the root exudate of sorghum and belonging to the family of benzoquinones. In *in vitro* assays, sorgoleone affects specific processes including photosynthetic and mitochondrial electron transport, while *in vivo* it is a potent inhibitor of PSII.^88^

Weston and Mathesius^89^ discuss the fact that autoallelopathy limits the renovation of pastures, since the high amount of phenolic acids and flavonoids released into the soil by the previous plant community can limit the germination and seedling growth of the next generation. Autoallelopathy and autotoxicity also result in replant issues. Yang *et al*.,^90^ for example, showed that the accumulation of ginsenosides produced by *Panax notoginseng* causes crop replant failure in continuously cultivated ginseng gardens.^90^ Autotoxicity bioassays showed that ginseng seedlings cannot survive in the presence of ginseng root extracts, soil on which ginseng was cultivated, or pure ginsenosides. As underlined by the authors, ginsenosides not only have an autotoxic effect, but also stimulate the growth of soil‐borne pathogens such as *Fusarium solani* and *Phytophthora cactorum*, which also contributes to replant failure.^90^

Allelopathy can also be a resource to protect crops against weeds, for example rice produces diterpene momilactones that suppress the growth of neighboring plants such as *Echinochloa crus‐galli* (barnyard grass), one of the rice paddy weeds that infest rice fields.^91^

### 4.7 Plant–nematode interaction

Plant pathogenic nematodes such as cyst and root knot nematodes need a host to complete their life cycle, hence they have adapted strategies to detect the presence of their host. Cyst nematodes belong to the families *Heterodera* and *Globodera* and attack many different plant species, including Solanaceae (potato, tomato and eggplant), sugar beet, wheat, rice and soybean.^92^ At the end of their life cycle these parasitic nematodes form a structure called cyst that is released into the soil and can contain over 200 eggs. The cyst is formed by the female body and protects the eggs against biotic and abiotic stresses for up to 20 years.^92^, ^93^ When a suitable host is nearby, the eggs hatch in response to hatching stimulants produced by the host roots, after which the juveniles penetrate the root and induce a feeding site.^94^ Devine *et al*.^95^ detected multiple hatching factors in potato root exudate while Byrne *et al*.^96^ suggested that the glycoalkaloid α‐solanine can act as a hatching stimulant or inhibitor depending on the concentration. The most effective hatching stimulants, however, are the nortriterpenoids eclepins that have been reported in several species.^97^ Soybean produces glycinoeclepin A,^98^, ^99^, ^100^ kidney bean glycinoeclepin B and C,^101^ and potato solanoeclepin A.^102^

Root knot nematodes belong to the genus *Meloidogyne* and parasitize the roots of nearly every species of higher plants, thus are considered the most damaging group of plant‐parasitic nematodes.^103^ At the site of infection they induce galls or root‐knots which affect the nutritional status of the plant causing yield losses and consequently a reduction in product quality.^103^ Volatiles produced by roots of *Capsicum annuum*, such as α‐pinene and limonene, elicited positive chemotaxis in *Meloidogyne incognita*, with methyl salicylate showing the highest effect in terms of attraction.^104^ Recently Čepulytė *et al*.^105^ found in tomato and *Medicago* root exudates from seedling root tips, powerful – non‐volatile – attractant(s) for three root‐knot nematode species, but could not identify them. It is unclear whether root‐knot nematodes use host‐specific cues or rather a non‐specific blend of volatile and non‐volatile compounds.

## 5 EXPLOITATION OF BELOWGROUND SIGNALING IN AGRICULTURE

Despite the numerous examples of signaling relationships in the soil that we described above, the majority of compounds that are secreted in the root exudates have no known function attributed to them, other than perhaps being a carbon source for microbes.^29^ We think that the latter is a gross underestimation of the importance of root exudates for plants. For those molecules that have been characterized and have at least one clear role in the rhizosphere attributed to them, challenges may remain, as demonstrated for the strigolactones for which it took 40 years to discover a second, beneficial, role after the discovery of their parasitic plant seed germination activity in 1965.^47^, ^106^ Finally, the translation of unambiguous results on biological activity in model or even *in vitro* studies to the field is challenging because soil is an unpredictable substrate, catalysing the degradation of organic molecules, it is not homogenous and it is subject to changes due to weather and other environmental factors such as flooding, agricultural practices and organisms living within it. Nevertheless, for some rhizosphere signaling molecules, research is going on into their potential to be exploited in agricultural systems. Signaling molecules that are present in plant root exudates can be exploited in different ways: through breeding,^107^ application of compounds,^108^ intercropping^109^ or crop rotation.^110^, ^111^, ^112^ We will discuss some examples of how these techniques can be applied and in which circumstances they can have drawbacks.

Breeding for the production (or lack thereof) of specific cues is an attractive approach to try to optimize rhizosphere interactions.^113^ North American maize lines do not produce (or produce very low amounts of) (*E*)‐β‐caryophyllene, and thus do not effectively attract entomopathogenic nematodes that can control the corn root worm. These lines have a functional (*E*)‐β‐caryophyllene synthase, but it is not expressed. Degenhardt *et al*.^114^ in 2009 showed how restoring the production of (*E*)‐β‐caryophyllene in these lines through transformation with the oregano (*E*)‐β‐caryophyllene synthase results in the (restored) attraction of entomopathogenic nematodes that parasitize and kill the larvae of the western corn rootworm. This resulted in a 60% reduction in adult corn rootworm occurrence in field experiments. Although the production of (*E*)‐β‐caryophyllene can likely also be restored through classical breeding, as the authors suggest, up to now this objective has not been achieved due to the long time needed for classical introgression of even single‐gene‐based traits.

Fernández‐Aparicio *et al*.^107^ showed that there is genetic variation in faba bean (*Vicia faba*) for broomrape germination stimulant production, offering the possibility to select for this trait in breeding programs. Similarly, Pavan *et al*.^115^ described the selection of a pea line (*Pisum sativum*), resistant to the parasitic weed *Orobanche crenata* through lower production of strigolactones. Although a lower production of strigolactones seems to be an advantage in these specific contexts, tomato lines in which strigolactone biosynthesis was reduced through an RNAi strategy displayed severely reduced stem height and increased shoot branching due to the reduced hormonal control by strigolactones, making this an unsuitable strategy for resistance breeding.^116^, ^117^

Beneficial compounds can also be applied directly to the soil. Rasmann *et al*.^81^ applied (*E*)‐β‐caryophyllene directly into the field and found a more than two‐fold decrease in western corn rootworm adult emergence. Similarly, Devine and Jones^108^ compared the hatching rate of potato cyst nematode in soil and *in vitro* after application of potato and tomato root leachate. They concluded that the direct application of a hatching stimulant (pure or in a mixture) can be used to induce egg hatching in the absence of a host, which would result in the death of the juvenile and a decrease in the PCN population, a procedure called ‘suicide hatch’ by Devine and Jones.^108^

This strategy has also been suggested for the prevention of parasitic weed infection.^118^ Indeed, synthetic strigolactone analogs sometimes display very high germination stimulant activity, but just as for natural strigolactones, the major challenge is their instability in soil and consequent decomposition. Encapsulation using specific formulations to deliver the product on the desired site, protect it against external agents and/or to improve its efficacy could be solutions to this problem. On the basis of these considerations, Zwanenburg *et al*.^119^ applied the strigolactone analog Nijmegen‐1 in the field, using a formulation that included an emulsifier, and obtained promising results.

Intercropping is an interesting strategy to interfere with rhizosphere signaling. The most intriguing example of this is the push–pull strategy developed by Khan *et al*.^109^ In their search for an effective control strategy of maize stem borers they serendipitously found that intercropping maize with the cattle forage legume *Desmodium uncinatum* reduced infection by the parasitic witchweed *Striga hermontica* and consequently increased maize yield. Hooper *et al*.^120^ showed that *D. uncinatum* exudes the C‐glycosylflavone isoschaftoside, which acts as an allelochemical and inhibits radicle growth of Striga, thus reducing and preventing maize parasitism.

Trap cropping is another technique that can be used to prevent infection of crops by pathogenic organisms. Scholte^110^ investigated this for potato cyst nematode and postulated that a good trap crop should stimulate hatching of juveniles by producing high levels of hatching stimulant and at the same time being resistant, not allowing infection or the development of the nematodes.^110^, ^121^ Scholte^110^ suggested *Solanum sisymbriifolium* as a trap crop to control PCN in a system of crop rotation to keep nematode populations at low levels, and Dias *et al*.^122^ suggested this species also for controlling other plant‐parasitic nematode populations.

## 6 FUTURE PERSPECTIVES FOR RHIZOSPHERE SIGNALING MOLECULES

Molecules released in the root exudates of plants can be exploited as antibiotics, allelochemicals, pathogen and pest repellents, and for improving nutrient availability and for their action as signaling molecules that attract beneficial organisms to the plant. Of utmost interest for agriculture are, for example, the phytoanticipins produced by plants that inhibit growth of and root colonization by pathogens. A better understanding of the efficacy of these molecules and their potential application in the field could perhaps (partially) replace the use of synthetic pesticides. The same consideration holds for allelopathic compounds that could be used in combination with or to replace herbicides in order to reduce the selective pressure on invasive species and other weeds when treated with the same chemical for long periods of time. From the perspective of IPM, using such new molecules could be advantageous, improving the quality of crops without having to use artificial chemistry. Of course, plant‐derived molecules could be as toxic as synthetic pesticides, and despite all the advantages that these molecules can provide, the normal procedures to assess their safety must be considered. Root exudate molecules can also help in the amelioration of the nutritional status of the plant, for example using organic acids and phytosiderophores in soils where there is poor nutrient availability or where nutrients are adsorbed to soil particles. The use of plant signaling molecules to stimulate beneficial interactions between plant roots and soil microorganisms, such as PGPBs and (other) symbionts, can lead to preventive protection of crops against pathogens by boosting the plant immune system. Such relationships can bring also nutritional advantages, for example AM fungi and rhizobia interact with plant roots in symbioses that improve the availability of phosphorous and nitrogen, respectively, for the plant.

Many of these beneficial relationships have been known for decades, but still very little is known about the signaling molecules that trigger the association between plants and microorganisms, or the signaling pathways that plants and soil organisms have evolved to perceive and respond to these cues. A better understanding of the mechanisms underlying the myriad interactions that take place in the plant rhizosphere and the signaling molecules produced by both parties could help us improve current agricultural practices. QS, for example, represents an intriguing area and has been intensively studied in microorganisms. The fact that plants produce QS‐mimicking molecules is highly intriguing and represents a potentially powerful tool to develop strategies against pathogens. Finally, more efforts are needed to find ways of exploiting these molecules for field application, since most of the current knowledge is based on fundamental research that ignores the challenges of scaling‐up to industrial application. A connection between fundamental and applied research is therefore needed to link the discovery of new molecules to their potential beneficial role in cultivation.
